# Ostial plication: a rarely reported cause of sudden death

**DOI:** 10.1186/1746-1596-5-15

**Published:** 2010-02-25

**Authors:** Fabio De-Giorgio, Vincenzo Arena

**Affiliations:** 1 Institute of Legal Medicine, Catholic University, School of Medicine, Rome, Italy; 2 Institute of Pathologic Anatomy, Catholic University, School of Medicine Rome, Italy

## Abstract

We report a rare case of ostial plication as a potential cause of sudden death. Very few reports and images are available in the specialized literature regarding this anomaly. Ostial plication may be a source of sudden death or cause of death when no other significant autopsy findings are present.

Ostial plication is a congenital severe obstruction/occlusion of the right or left ostium. Plication of the aortic wall leads to a "valve-like" ridge that can act as a door blocking inflow during diastolic filling, with consequent ischemia and a potentially life-threatening arrhythmia. The true incidence of this condition and its relationship to sudden death have not been reported in the literature. We believe that this case will be useful to autopsy pathologists in detecting this infrequent anomaly, thus providing a more accurate estimation of its incidence.

## 

Coronary artery anomalies (CAAs) can be classified on the basis of their origin, course, size, and termination [1-3].

Although general agreement exists of the potential relationship between CAAs and sudden death, the true incidence and pathogenesis, especially in sudden unexpected non-traumatic death in a young adult population (aged 20-40 years), have not been well defined.

Ostial plication is a rare congenital abnormality comprising severe obstruction/occlusion of the right or left ostium. Plication of the aortic wall leads to a "valve-like" ridge that can act as a door blocking inflow during diastolic filling. Consequent ischemia may produce a life-threatening arrhythmia [4,5]. According to Virmani et al. [5], the surface area of the ridge must exceed 50% of the coronary ostial lumen to be related to sudden death.

The true incidence of this condition and its association with sudden death have not been reported in the literature.

We present a 30-year-old woman who was found unconscious in bed by her husband. She was taken to hospital, but pronounced dead on arrival. According to her family, her past medical history was unremarkable. Her body was transported to our Institute, where a post-mortem examination was performed. The post-mortem toxicological examination, including a general drug screening, was negative. At autopsy, abnormal autopsy findings were limited to the heart, which weighed 275 grams. The myocardium had focal areas of sclerosis in the left ventricular antero-lateral wall and signs of recent ischemia. The valves were normal and the right and left coronary artery ostia originated unremarkably from the aorta. Interestingly, the left coronary ostium showed a reduction of its lumen due to an ostial plication > 50% (ostium diameter = 4.70 mm).

The plication occluded the lumen in a "fold-like"manner [Figure 1].

**Figure 1:**
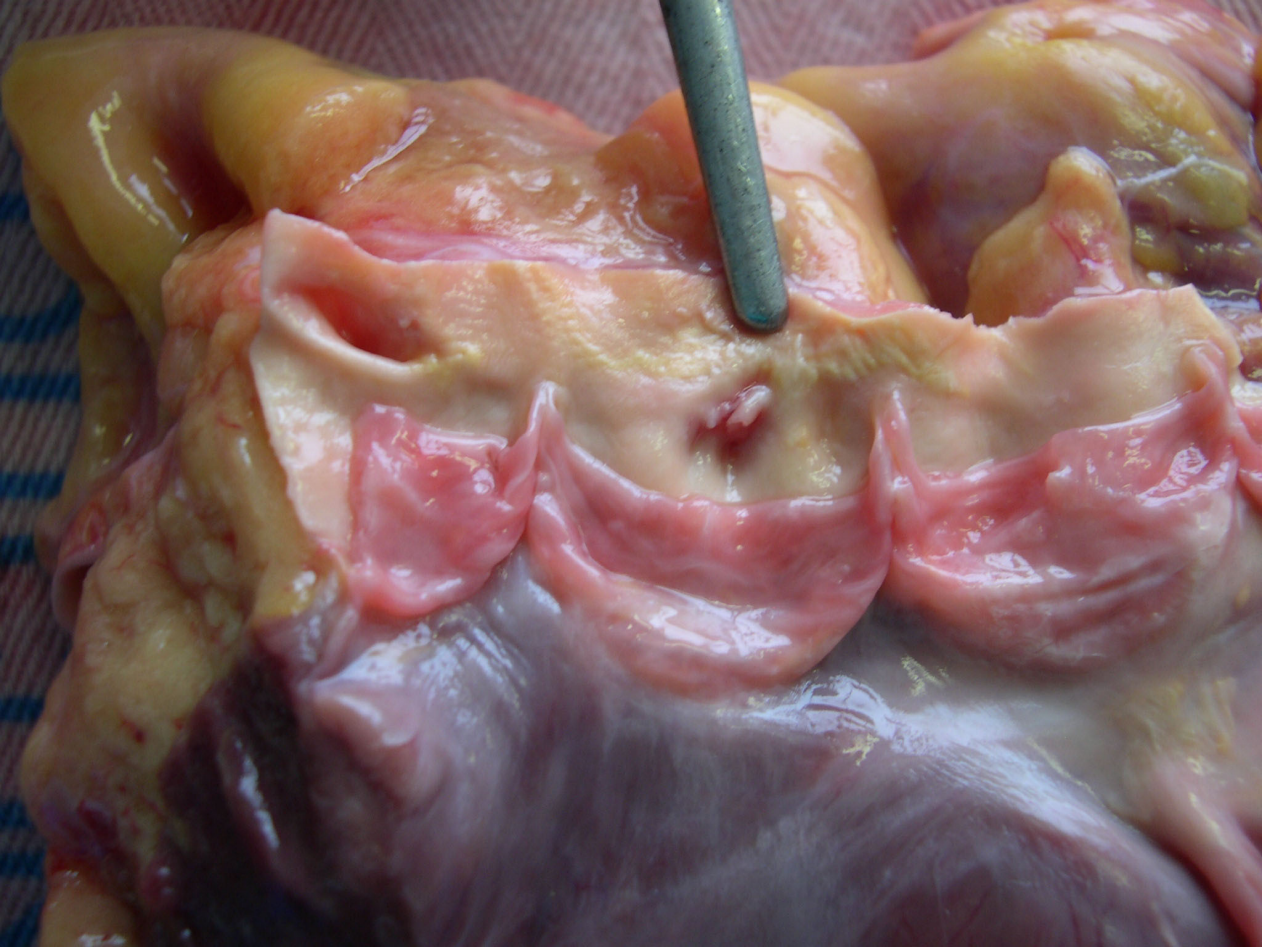
**Macroscopic image of the left coronary ostium showed a reduction of its lumen due to an ostial plication occluding the lumen in a "fold-like"manner**.

No coronary course anomalies were found.

Histological examination of the tributary myocardial wall supplied by the left coronary artery confirmed signs of chronic ischemia, with scarring and mild myocyte hypertrophy. The surrounding myocardium showed signs of recent ischemic damage, reflected by myocyte ballooning and waving.

In conclusion, ostial plication is a potential source of sudden death or cause of death that should be considered when no other significant autopsy findings are present.

We believe that this case will be useful to autopsy pathologists in detecting this infrequent anomaly, thus providing a more accurate estimation of its incidence.

## Competing interests

The authors declare that they have no competing interests.

## Authors' contributions

FDG performed the forensic autopsy. FDG and VA participated in the design of the study. Both authors read and approved the final manuscript.
